# Accelerated identification of disease-causing variants with ultra-rapid nanopore genome sequencing

**DOI:** 10.1038/s41587-022-01221-5

**Published:** 2022-03-28

**Authors:** Sneha D. Goenka, John E. Gorzynski, Kishwar Shafin, Dianna G. Fisk, Trevor Pesout, Tanner D. Jensen, Jean Monlong, Pi-Chuan Chang, Gunjan Baid, Jonathan A. Bernstein, Jeffrey W. Christle, Karen P. Dalton, Daniel R. Garalde, Megan E. Grove, Joseph Guillory, Alexey Kolesnikov, Maria Nattestad, Maura R. Z. Ruzhnikov, Mehrzad Samadi, Ankit Sethia, Elizabeth Spiteri, Christopher J. Wright, Katherine Xiong, Tong Zhu, Miten Jain, Fritz J. Sedlazeck, Andrew Carroll, Benedict Paten, Euan A. Ashley

**Affiliations:** 1 Stanford University, Stanford, CA USA; 2 UC Santa Cruz Genomics Institute, Santa Cruz, CA USA; 3 Stanford Health Care, Palo Alto, CA USA; 4 Google Inc, Mountain View, CA USA; 5 Oxford Nanopore Technologies, Oxford, UK; 6 NVIDIA Corporation, Santa Clara, CA USA; 7 Baylor College of Medicine, Houston, TX USA

**Keywords:** DNA sequencing, Genetics research, Computational biology and bioinformatics

## Abstract

Whole-genome sequencing (WGS) can identify variants that cause genetic disease, but the time required for sequencing and analysis has been a barrier to its use in acutely ill patients. In the present study, we develop an approach for ultra-rapid nanopore WGS that combines an optimized sample preparation protocol, distributing sequencing over 48 flow cells, near real-time base calling and alignment, accelerated variant calling and fast variant filtration for efficient manual review. Application to two example clinical cases identified a candidate variant in <8 h from sample preparation to variant identification. We show that this framework provides accurate variant calls and efficient prioritization, and accelerates diagnostic clinical genome sequencing twofold compared with previous approaches.

## Abstract (web summary)

A streamlined sequencing process enables identification of disease-causing variants in the clinic within 8 hours.

## Main

WGS harbors advantages for medical diagnosis^1,2^, especially in a critical care setting, but pipelines for sequencing and downstream analysis have typically been slow^3,4^. One approach based on short-read sequencing initially reported a time for diagnosis of 56 h^5^. By reducing the compute time for this same sequencing approach, we reported a pipeline capable of making a diagnosis in 48 h^6^. In 2019, it was reported that developments to this pipeline returned results in as little as 19:10 h (19:10–31:02 h)^7^. In 2021, one case was reported at a turnaround time of 14:33 h^8^.

With recent advances, nanopore sequencing has emerged as a high-throughput, high-fidelity sequencing platform^9,10^. We speculated that, with a capacity for 48 flow cells, the PromethION platform (Oxford Nanopore Technologies) would have the ability to sequence a single sample to a clinical quality depth in 2 h. Furthermore, we hypothesized that alignment, variant calling and variant filtration could be started in real time and completed within hours. However, several technical challenges were immediately apparent in realizing this vision. First, conventional sample preparation protocols do not account for the generation of sequencing libraries sufficient for the rapid clinical use case from a limited volume of blood, something particularly relevant when samples are derived from critically ill neonates. Second, although complete reads can be streamed off the nanopore sequencing device in real time and within minutes of starting a run, with 48 flow cells running in parallel, the rate of data production far outpaces the highest rate of base calling and alignment possible on the local PromethION compute tower (Data Acquisition Unit accompanying the Sequencing Unit). This leads to a high compute latency, where the base calling and alignment runtime are an order of magnitude higher than the sequencing time. Third, although small-variant, nanopore-calling pipelines have been shown to harbor high accuracy in gold-standard cell-line data^11^, their performance has not yet been characterized in clinical samples. Finally, traditional variant filtration and prioritization methods result in a high number (~100) of variant candidates that can be time prohibitive for the manual curation and confirmation required in a clinical setting.

In the present study, we address these challenges developing a whole-genome nanopore-sequencing pipeline with improvements in library preparation, a cloud-based module to perform near real-time base calling and alignment, accelerated variant calling (single nucleotide polymorphisms (SNPs), insertions and deletions (indels) and structural variants (SVs)) and focused variant filtering. We demonstrate the characteristics of this pipeline using the Genome In A Bottle (GIAB) HG002 sample^12^. Finally, we apply the pipeline to the diagnosis of a critically ill 57-year-old man and a 14-month-old infant, surfacing in both cases a candidate variant in <8 h after the blood draw, representing a 46–50% improvement on the fastest reported time (sample preparation to variant identification) to date^8^.

**Fig. 1:**
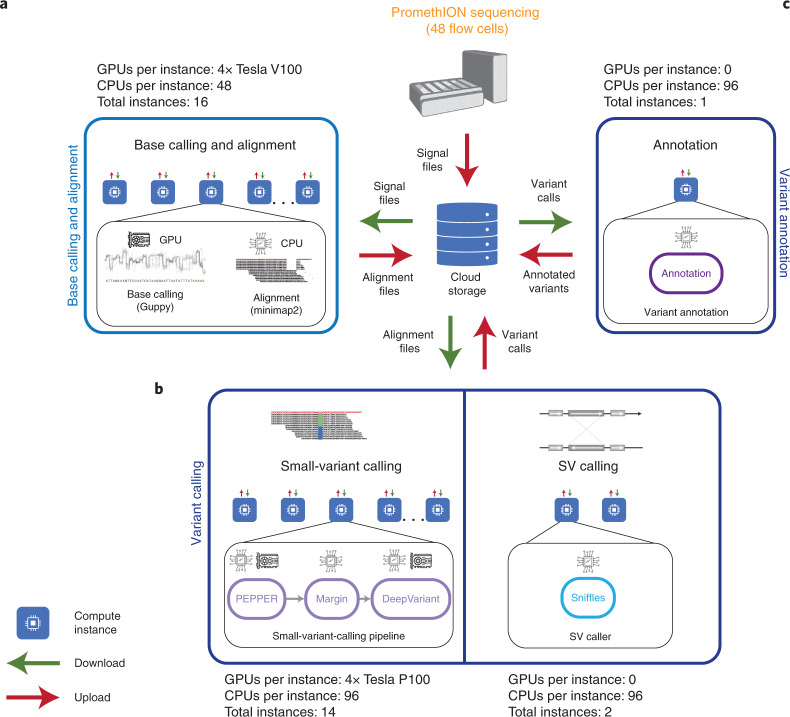
Overview of ultra-rapid computational pipeline. **a**, After the start of sequencing on the PromethION48 device, raw signal files are periodically uploaded to cloud storage. Our cloud-based pipeline scales compute-intensive base calling and alignment across 16 instances with 4× Tesla V100 GPUs each and runs concurrently with sequencing. The instances aim for maximum resource utilization, where base calling using Guppy runs on GPU and alignment using Minimap2 (ref. ^17^) runs on 42 virtual CPUs in parallel. **b**, Once the alignment file is ready, small-variant calling performed using GPU-accelerated PEPPER–Margin–DeepVariant^11^ on 14 instances and SV calling using Sniffles^18^ on 2 instances. Each instance processes a specific set of contigs. Specific details about the Google Cloud Platform-based instance configurations are provided in Supplementary Table 21. **c**, These variant calls are annotated to aid in the subsequent variant filtration and prioritization. Our score-based variant filtration method takes in millions of variants reported by the variant caller to surface any deleterious variant for review using Alissa. The filtration method is designed such that it reports a tractable number of variants for manual curation.

## Results

### Ultra-rapid, whole-genome, nanopore-sequencing pipeline

First, we adapted the standard sample preparation protocols to allow for a sufficient quantity of sample library to be distributed across 48 flow cells. To accommodate neonates and infants, we needed a DNA-extraction protocol to yield sufficient high-quality genomic DNA from a limited volume of blood. We tested multiple methods (Supplementary Table 1) and found an approach capable of isolating high-molecular-mass DNA with an average fragment size >60 kb, measured with electrophoresis (Tapestation, Agilent Technologies Inc.), a minimum of 36 μg of genomic DNA measured by fluorometry (Qubit, Invitrogen/Themo Fisher Scientific) and sample purity with an average of 1.70 measured by the 260-/280-nm ratio spectrophotometer (Nanodrop, Thermo Fisher Scientific) from 1.6 ml of blood in 50 min (PureGene, QIAGEN). Preparing a sequencing library for each of the 48 flow cells was not only time prohibitive, but would also require a total of 48 μg (1 μg per reaction) of starting DNA. Instead, we investigated whether we could exceed the recommended amount of input DNA per reaction while scaling down the number of library reactions. We found that increasing the input DNA to 4 μg per reaction, and preparing eight reactions in parallel, results in an optimal library yield of 16 μg, allowing for up to 333 ng of library to be loaded per flow cell.

**Fig. 2:**
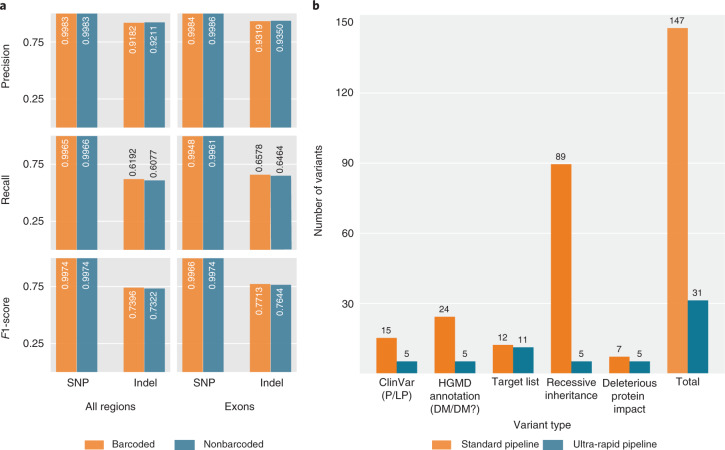
Comparison of barcoded and nonbarcoded variant calling performance, and standard and ultra-rapid variant filtration performance. **a**, Stratified variant calling performance comparison between barcoded and nonbarcoded HG002 samples in all benchmarking and exonic regions. The HG002 nonbarcoded run was the seventh sample run on the flow cells. The similarity in variant calling performance shows that barcoding is not necessary to achieve high-quality variant calls. **b**, Comparison between standard and ultra-rapid variant filtration pipelines. The standard pipeline picks variants if any of the subcategories are true for any variant, whereas the ultra-rapid pipeline uses a score-based method to surface mostly relevant variants for fast manual review. We see that the standard pipeline flags 147 variants compared with 31 variants proposed by the ultra-rapid pipeline, which substantially reduces the time required for manual review. Source data

To reduce per-sample cost, we re-used a set of 48 flow cells for multiple samples. To re-use the flow cells for consecutive samples, we removed the DNA library after each sequencing run with a standard nuclease wash as described in Online Methods. One approach to further minimize carryover from a previous library involves preparing each library with unique nucleotide sequences (barcodes) ligated to the genomic DNA. However, we observed that barcoding adds time and reduces the amount of DNA available to load per flow cell, due to an additional library cleanup needed for the barcoding protocol. Hence, we investigated whether barcoding was necessary for robustness in the pipeline. To assess the effect of barcoding on downstream variant calling performance, we measured carryover rates between samples, finding a maximum rate of 0.4% (Online Methods). We then modeled carryover at a higher rate by randomly introducing 1% HG005 reads to an HG002 sample (Supplementary Notes). We compared the variant call performance between the artificial sample with carryover and a pure sample, and found that there were no notable differences (Supplementary Table 2). These data suggest that at least 1% carryover is tolerated in our variant calling pipeline. For further validation, we sequenced the NIST reference material HG002 genome from the Personal Genome Project^13^, with and without barcoding on flow cells that had previously sequenced six different samples, each with similar throughput (Supplementary Table 3). The nonbarcoded VCF was generated incorporating all passed reads (strand *q* score ≥7), whereas the barcoded VCF was generated using only passed reads with the appropriate barcode. We then investigated whether the carryover in the nonbarcoded samples impacted variant calling by comparing variant call performance of each sample against NIST GIAB HG002 small-variant benchmarking data^14^. Figure 2a illustrates that HG002 variant calling performance with and without barcoding is similar (barcoded *F*1 score: 0.9974 SNP, 0.7396 indels; nonbarcoded *F*1 score: 0.9974 SNP, 0.7322 indels). Moreover, variant calling performance in stratified regions such as exons and complex regions of the genome is similar between barcoded and nonbarcoded data (Fig. 2a, Supplementary Fig. 1 and Supplementary Table 4). Similarly robust outcomes were observed in the barcoded and nonbarcoded SV calls when compared against the GIAB HG002 benchmarked SV data^15^ (barcoded *F*1 score: 0.915; nonbarcoded *F*1 score: 0.910; Supplementary Fig. 2). Based on these data, we elected to continue without barcoding. This resulted in a 37-min reduction in total library preparation time and improved downstream sequencing efficiency^16^.

Next, we addressed the limitation of the local compute tower with respect to real-time base calling and alignment. When running 48 flow cells and high-accuracy base calling models, the compute tower performs base calling and alignment at a slower rate than the maximum sequence data generation rate of 1.8 Gb min^−1^ (ref. ^16^). We established a baseline for running base calling (Guppy v.4.2.2) and alignment (Minimap2 v.2.17 (ref. ^17^)) sequentially on the local tower using the 218-Gb dataset generated from the earlier nonbarcoded HG002 sample. In this test, base calling ran for 17.5 h and alignment was completed in another 2.5 h. With a theoretical maximum throughput of 2.5 Gb min^−1^ across 48 flow cells, sequencing would run for 1.5 h and running base calling and alignment concurrently with sequencing would still leave us with a substantial 18.5-h overhead time (we define overhead time as the runtime for base calling and alignment after sequencing has been completed). To address this issue, we developed a cloud compute infrastructure (specifically, using Google Cloud Platform in our study) and parallelized base calling and alignment across multiple graphics processing unit (GPU) instances (each with four NVIDIA V100 GPUs). Although this configuration created an additional network memory bandwidth-intensive step of transferring terabytes of raw signal data from the tower to cloud storage, we solved this bottleneck by using improved fast5 (raw signal file type) file compression (VBZ), adjusting the number of reads per fast5 file and shifting to a timed periodic upload model. The upload management is coordinated with distribution of the raw data to 16 compute instances (Fig. 1a), each running Guppy and Minimap2 for a specific set of three flow cells per instance. Using this approach, we were able to achieve near real-time base calling and alignment at scale. We ran a simulation where all the fast5 files from the HG002 sample were randomly distributed into 48 subsets, each representing the throughput from a distinct flow cell. To simulate a throughput rate as high as 2.5 Gb min^−1^, the data from each subset were transferred at a uniform rate over a period of 90 min to a different output directory (consistent with the associated directory generated during every sequencing run). With 16 instances running in parallel, the base called and aligned output files were generated with an overhead time of 25 min. In this way, base calling and alignment of a high-depth (200 Gb), long-read, whole human genome can be completed in near real time.

Next, we approached the acceleration of variant calling. We used PEPPER–Margin–DeepVariant^11^ to identify small-variants and Sniffles^18^ for SV calling. We scaled variant calling to several cloud instances to achieve runtime acceleration. We used 14 instances with GPU for PEPPER–Margin–DeepVariant and 2 central processing unit (CPU)-only instances for Sniffles (Fig. 1b). Each small-variant calling instance processes a single contig or a pair of assigned contigs sequentially (Supplementary Table 5). To derive the contig distribution, we first profiled the variant calling runtime for each contig with the HG002 sample (Supplementary Table 6). As PEPPER–Margin–DeepVariant uses long-range phasing information during variant calling, we did not go below chromosome scale for parallelization. Similarly, we sped up Sniffles by running genomic sections in parallel (Supplementary Table 7) with a varying number of threads, resulting in a runtime of 29 min for the HG002 sample. We balanced sensitivity to larger duplications and inversions and interchromosomal translocation events in the choice of chromosome-level structural variant calling.

As a proof-of-concept, we demonstrated a further reduction of overall runtime from 40 min to 23 min by integrating the GPU-accelerated implementation of DeepVariant available with the NVIDIA Parabricks toolkit (https://www.nvidia.com/en-us/clara/genomics) into the PEPPER–Margin–DeepVariant pipeline. It reduced the overall runtime from 40 min to 23 min (Supplementary Table 6). Although several previous studies have demonstrated the performance of the nanopore-based variant calling to be competitive against other methods^14,19–21^, we further improved the variant calling accuracy of our pipeline. The main error mode of nanopore sequencing is indels, especially in homopolymers. To improve the accuracy of indel calling and thereby reduce curation time, we modified the DeepVariant pile-up image to include a realignment of reads to the alternative allele of an indel event, a process first described for long-read sequencing for the Pacific Biosciences platform (https://ai.googleblog.com/2020/09/improving-accuracy-of-genomic-analysis.html). This expands insertion sequences and improves generalization by making the alignment support for the alternative allele look similar to real events of either deletions or insertions. As a result, our indel *F*1 score increased from 0.6999 to 0.7322 (Supplementary Fig. 3). Specifically, the total number of reported variants decreased by 3% (from 4,439,940 to 4,308,281), whereas we observed increased accuracy, thereby reducing the curation time required to assess potential variants (Supplementary Table 8).

Last, we annotated the variant calls (Fig. 1c) and developed a customized schema for variant filtration to accelerate manual review of variants. For each sample, we derived a patient-specific, phenotype-based target gene list in collaboration with treating clinicians. The small-variant vcf file was analyzed using Alissa Interpret (Agilent Technologies Inc.) and variants were filtered and prioritized for review using a customized classification tree. The customized classification tree is an adaptation of Stanford Clinical Genomics Program’s (GCP’s) clinically validated proband exome classification tree (Online Methods). This standard filtration scheme is designed for application to patients on a diagnostic journey and provides a broad search that allows for phenotype expansion as well as limited gene–disease discovery. In the standard system, manual review is triggered when the variant meets any one of several possible criteria (or sets of criteria), including previous annotation, presence on a patient-specific gene list and potential biallelic inheritance or predicted deleterious impact (Supplementary Table 9). For application in the rapid setting, our goal was to readily surface clearly pathogenic, actionable variants in established disease genes. The same categories of criteria were applied as in the standard system, but, instead of acting as a trigger, each criterion was scored independently (Supplementary Table 10) and manual review was implemented only if the total score met our empirically derived threshold of ≥4. In addition, many criteria were evaluated under tighter constraints for the rapid system. For example, in the standard system, all rare biallelic variants were evaluated; in the rapid system biallelic variants received points only if those genes were associated with autosomal recessive disease in OMIM (Online Mendelian Inheritance in Man)^22^. In the case of the above HG002 sample, the prioritized variant count was reduced from 101 in the standard system to 20 for the rapid approach (Supplementary Table 8).

### Pipeline application and performance in a clinical setting

**Fig. 3:**
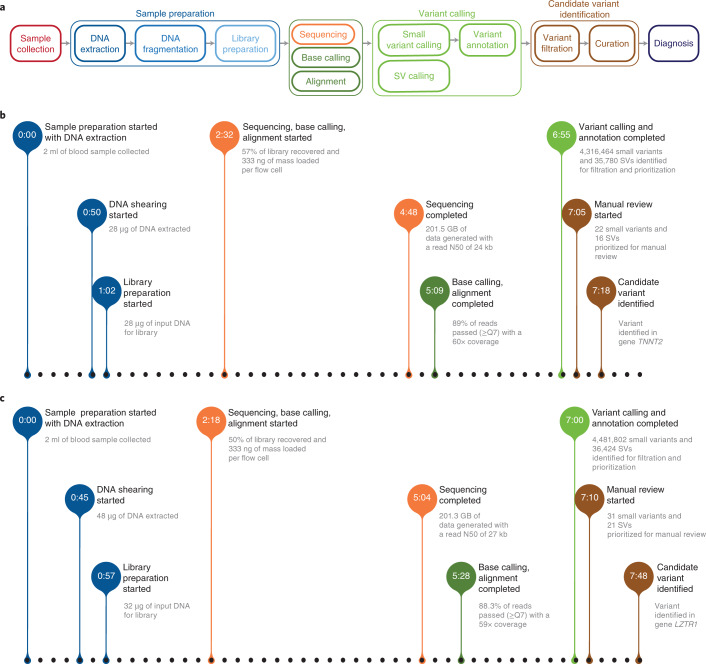
Ultra-rapid-sequencing pipeline performance. The detailed schema of the ultra-rapid-sequencing pipeline and end-to-end performance of the ultra-rapid-sequencing pipeline on two different clinical samples. **a**, The ultra-rapid-sequencing pipeline starts from sample collection on the far left to final diagnosis on the far right. The details of each step are presented in Online Methods. All the steps that run in parallel are vertically stacked. **b**, The first patient was the fourth sample sequenced on the set of flow cells, resulting in 2:16 h of sequencing. Variant calling completed 6:55 h from the start of sample preparation. Subsequently, variant filtration and manual review identified a candidate variant in gene *TNNT2* in 7:18 h. **c**, The second patient was the sixth sample on the same set of flow cells. The sequencing completed in 2:46 h with 200 Gb followed by another 2:44 h to identify a candidate variant resulting in an end-to-end time of 7:48 h.

To illustrate the performance of this pipeline (Fig. 3a) in a real-world setting, we summarized the clinical presentation and the pipeline details for two cases. First, a 57-year-old man with a severe SARS-CoV-2 infection and comorbidities, including hyperthyroidism and hypertension, required a bilateral lung transplantation. Intraoperative transesophageal echocardiography revealed biventricular dysfunction with left ventricular hypertrophy and postoperative episodes of sinus bradycardia. A cardiac magnetic resonance image provided evidence of hypertrophic cardiomyopathy; however, the differential diagnosis was wide and included coronary artery disease, myocarditis, cardiac amyloid and cardiac sarcoid. Rapid molecular testing was requested to help clarify the diagnosis. In 6:55 h (Fig. 3b), variant calling resulted in 4,316,464 small-variants (Supplementary Table 11) and 35,780 structural variants. In 7:18 h from the start of sample preparation, a diagnostic heterozygous variant (approximately 341 C > T) was identified in the *TNNT2* gene and classified as likely pathogenic according to the American College of Medical Genetics and Genomics (ACMG) guidelines^23^. The diagnosis further reduced the need for multiple follow-up imaging studies and cardiac biopsy.

Second, we identified a 14-month-old female infant with a history of dystonic/opisthotonic posturing and developmental delay, who was admitted to the pediatric intensive care unit at Stanford’s Lucile Packard Children’s Hospital after experiencing a cardiac arrest and respiratory failure. Brain magnetic resonance imaging revealed generalized ventricular prominence, a small pons and a thin corpus callosum. All other diagnostic testing was unremarkable, suggesting the possibility of a genetic etiology. The sequencing time to generate 200 Gb was 2:46 h (1.2 Gb min^−1^, lower than our peak throughput rate of 1.8 Gb min^−1^). In 7 h (Fig. 3c) from the blood draw, 4,481,802 small-variant calls (Supplementary Table 11) and 36,423 SV calls were generated. The filtration method surfaced 31 small-variants and 21 SVs prioritized for manual review and, within the next 48 min, we identified a candidate variant of unknown significance (approximately 791 + 1 G > A) in *LZTR1*, a gene suspected of being involved in the stabilization of the Golgi complex. This variant was manually reviewed and ultimately any pathogenic contribution was determined to be unclear. No other prioritized variants were suspected to contribute to the patient’s clinical presentation. Orthogonal clinical genetic testing did not identify a genetic etiology. Family-based studies are ongoing to further characterize any potential contribution of this variant.

We separately examined the effectiveness of our filtration tree, comparing the numbers of variants marked for review by the standard system with those marked for review by the rapid system (Fig. 2b) for patient 2. The standard system prioritized 147 variants for review. The rapid system scored 209 in total, but 178 variants had total scores of 1–3 and so did not meet the threshold for review. As noted above, a total of 31 variants were marked for review by our system, enabling rapid completion of the manual curation aspect of our pipeline (Supplementary Table 8).

## Discussion

The need for rapid clinical diagnosis from WGS is pressing^24,25^. Although the standard turnaround time for clinical WGS is weeks, recent studies have reduced the turnaround times in neonatal populations to a range of 3–5 d^26–28^. In the present study, we developed a streamlined approach to nanopore WGS that provides accurate small and large variant calls faster than any previously reported clinical WGS pipeline. The pipeline is capable of generating high-depth, human whole-genome data in <2 h and diagnostic variant calls in <8 h. This pipeline has been shown to be up to 50% faster than the previously reported fastest genome diagnosis made in 14:33 h^8^. Overall, this pipeline has been shown to be associated with a 42% diagnostic rate^16^.

Our approach to sequencing a single human genome across 48 nanopore flow cells required the solving of several technical challenges. During sample preparation, we focused on maximizing DNA quality and length while limiting the preparation time, especially for small volumes of blood. Compute methods were adapted to reduce runtime by the use of massive parallelism in the cloud and GPU acceleration. Although our study used Google Cloud Platform, the pipeline can easily be adapted to run on other cloud platforms as well. Other optimizations in speed or accuracy create trade-offs. For example, the additional alignment of the alternative allele results in more accurate but slower variant calling, although it speeds variant prioritization by reducing the number of frameshift variants for inspection. Another example not explored in the present study is the use of more accurate, but slower, base calling software. As infrastructure to scale computation unambiguously reduces runtime, in theory it is possible to use greater parallelism to further mitigate trade-offs of runtime for accuracy in the analysis methods.

The nanopore-based method can generate high-quality variant calls outside low-complexity regions^11,19^ and within medically relevant genes^20,29^. It has also been shown that outside the GIAB high-confidence region, nanopore-based variant calls have high Mendelian consistency^21^. Previous studies also show that variant calling performance (*F*1 score) of nanopore in Gencode v.35-annotated coding sequences in the human genome is 0.998 for SNPs and 0.858 for indels compared with PacBio HiFi’s 0.999 for SNPs and 0.945 for indels^11^, and Illumina’s 0.994 for SNPs and 0.992 for indels (Supplementary Table 12). These stratified analyses suggest that nanopore can identify pathogenic variants in coding sequences with high accuracy. In addition, a variant calling performance analysis using the Human Gene Mutation Database (HGMD)^30^ database (Online Methods) in Supplementary Table 13 shows that the nanopore pipeline has an SNP recall of 0.995, comparable to Illumina’s recall of 1.00. However, the nanopore pipeline has a lower indel recall of 0.68 compared with Illumina’s indel recall of 0.96. Nanopore-based methods are error prone in low-complexity regions with homopolymers, in particular indel variants in homopolymer regions. In extending this technology to medical-grade application, we designed a variant curation scheme that flags calls in homopolymer regions to lower their priority during the review process. Moreover, with the improvement in the nanopore technology such as R10.4 chemistry and more accurate base calling, we would expect substantial improvement in the nanopore indel performance in the near future.

One aspect of this pipeline is to use phase information to improve variant calling. The value, particularly for identifying false-positive calls, was readily apparent across the clinical cases on which this pipeline has subsequently been tested^16^. Gains are substantial, particularly for large and complex genes. A further advantage of nanopore sequencing is the ability to derive methylation information directly from the raw signal. Although methylation changes as a cause of genetic disease are uncommonly recognized, literature reports^31,32^ are probably an underestimate of the overall incidence because technology to quantify genome-wide methylation has, to date, remained mostly in the research domain.

Variant databases such as gnomAD^33^ contain information about variant frequencies across large study populations. However, such databases were largely constructed using short-read sequencing technology and are therefore biased toward point variations and regions accessible to short-read mapping. As adoption of long-read technologies increases, these limitations will be reduced, speeding prioritization by more comprehensively annotating common variants. In addition, trio approaches^26^ have known advantages in diagnostic sensitivity that have been well documented. Although we would expect trio sequencing to increase our yield, the yield currently presented (42%)^16^ is very much in line with the yield of genome assays presented over the last decade, suggesting excellent overall performance with the balance of enhanced SV detection and increased false-positive indel reporting in low-complexity regions.

In summary, we present a pipeline for high-depth nanopore sequencing of a human genome in <2 h combined with real-time base calling, alignment and accelerated variant calling and filtration, allowing the surfacing of candidate genetic variants in <8 h.

## Methods

### Samples and sample collection

- **Standard** HG002: cell pellets from HG002 cell line were a generous gift of M. L. Salit. HG002 and HG005 data for the in silico carryover experiment were obtained from the dataset provided in ref. ^11^.
- **Patient sample**: the patients presented in this text were recruited to our study because their clinical presentation was suspected to have a genetic etiology. We acquired consent from adults directly and, for minors, from parents or guardians according to the Stanford Institutional review board protocol 58559.

### Sample preparation

- **DNA extraction:** we extracted high-molecular-mass DNA (hmwDNA) from 1.6 ml of whole blood in EDTA using a modified protocol for the QIAGEN Puregene kit. First, red blood cells were lysed by adding blood to red cell lysis buffer at a 1:3 ratio and incubating for 5 min at room temperature. Nucleated cells were then isolated by centrifuging the solution at 15,000*g* for 30 s. The pelleted cells were lysed using cell lysis solution and proteinase K, and incubated at 56 ^∘^C for 5 min. Protein was then precipitated and removed using protein precipitation buffer and incubated on ice for 5 min, followed by a 1-min centrifugation at 15,000*g*. The supernatant was then recovered and incubated on ice for an additional 5 min and centrifuged for 3 min at 15,000*g* to remove any residual protein. DNA was precipitated in isopropanol and pelleted by centrifugation for 1 min at 15,000*g*. DNA was washed with cold 70% ethanol and re-pelleted by centrifugation for 1 min at 15,000*g* and then dried for ~2 min at 37 ^∘^C and resuspended in ultrapure water.
- **Fragmentation:** hmwDNA was sheared using Covaris g-tubes. In short, 50 μl of hmwDNA 80 ng μl^−1^ (4 μg) was applied to the g-tube and centrifuged at 1,450*g* in 1-min increments until the entirety of the DNA solution passed through the tube. The tube was then inverted and the process repeated. If the yield of hmwDNA permitted, this process was done in multiples of 8 to result in a 32-μg pool of fragmented DNA.
- **Library preparation:** standard sequencing libraries were prepared using the SQK-LSK109 kit (ONT). Barcoded sequencing libraries were prepared using the SQK-LSK109 and EXP-NBD104 (ONT). Libraries were prepared in 8× reactions according to the manufacturer’s protocol with multiple alteration. Input-sheared DNA was increased to 4 μg per reaction and repaired with formalin-fixed paraffin embedding (New England Biolabs), end-prepped with Ultra II End-Prep (New England Biolabs) and incubated at 20 ^∘^C for 5 min and then 65 ^∘^C for 5 min. This reaction was cleaned up using AmpureXP Beads (Agencourt) and washed with 70% ethanol. Libraries designated for barcoding had ONT native barcodes ligated in the following reaction: 24 μl of recovered DNA from end-prep, 5 μl of NBD and 29 μl of Blunt/TA Ligase Master Mix (New England Biolabs), and incubated at room temperature for 10 min. This reaction was then cleaned up using AmpureXP beads and washed with 70% ethanol. Barcoded libraries then had ONT adapters ligated using AMII (ONT), Quick T4 DNA ligase and NEBNext Quick Ligation Reaction Buffer. Nonbarcoded libraries had ONT adapters ligated using AMX (ONT), Quick T4 DNA ligase (New England Biolabs) and LNB (ONT). Libraries were cleaned up after adapter ligation with AmpureXP beads (Agencourt) and washed with long fragment buffer (ONT). After each cleanup step, libraries were pooled and DNA was quantified using a Qubit.
- **Flow cell preparation and sequencing:** 48 R9.4.1 flow cells (product code FLO-PRO002) were brought to room temperature, loaded into PromethION48 (ONT) and checked for available pores. Each flow cell was primed with 500 μl of priming buffer (FB + FLT) twice, separated by an incubation period of approximately 20 min. The pooled sequencing library was mixed with SQB and LB (ONT) and was equally distributed over the flow cells.

Complete details of the protocols are open sourced^34^ under the Creative Commons Attribution License.

#### Carryover rate after nuclease wash

As a set of 48 flow cells was used for multiple samples, we investigated the fraction of reads carried over from a previous library (carryover rate) by preparing 12 libraries, loading a set of flow cells with a single library, sequencing for a total of 90 min and then performing a nuclease wash. We repeated this process for each of the 12 libraries on the same flow cells. We filtered out the reads with a *q* score <7 (‘failed’ reads) and the reads that were not mapped to any barcode (unclassified reads) (Supplementary Table 14). We then determined the carryover rate by calculating the fraction of reads with barcodes that did not match the expected barcode for each sequencing run (Supplementary Fig. 4). The highest rate of carryover observed was 0.4%.

#### In silico carryover experiment

In our experimental setup to test the robustness of variant calling performance with and without barcoding in silico, we considered HG005 (Chinese son) as patient 1 and HG002 (Ashkenazi son) as patient 2. The pure HG002 sample data are analogous to a barcoded sample and the HG002 sample with 1% carryover from HG005 represents the nonbarcoded sample. We used two different individuals of different ancestry to the two patients to estimate the effect of carryover rate. We took 1% reads from HG005 and added to HG002 data to simulate the carryover from one sample to the other.

### Sequencing, base calling and alignment

Once sequencing starts, fast5 files (raw signal files) are uploaded to a cloud storage bucket every 3 min using the *cron* utility and *rsync* utility from the Google Cloud SDK for synchronizing and avoiding any redundancy in the upload. The raw bandwidth requirement is improved by configuring VBZ compression of the fast5 file. There is a trade-off between reducing the total latency associated with the application programming interface (API) calls for data transfer (improved by increasing file size) and increasing the number of files that can be uploaded simultaneously. Hence, we set the number of reads per fast5 file to 10,000 and used VBZ compression for the signal data (https://github.com/nanoporetech/vbz_compression.git) that resulted in a fast5 file size of around 1 GB for near real-time data transfer.

We used Guppy v.4.2.2 for base calling the sequenced reads and Minimap2 v.2.17 (ref. ^17^) to align the base called reads to the GRCh37 human reference genome using 16 instances. Every instance uses a cron job each, for base calling and alignment. For base calling, the cron job first checks whether the previous job was running; if not, the new batch of fast5 files in cloud storage data generated from three specific flow cells (Supplementary Table 15) assigned to the instance are downloaded and base called. We used the high-accuracy model for base calling, along with the corresponding configuration provided for acceleration using NVIDIA Tesla V100 GPUs. Reads with a *q* score <7 (assigned ‘fail’ by Guppy) are filtered out and reads that pass the threshold constitute an alignment job for the batch. If a previous alignment job is not running, a new alignment job from the queue is started. Once sequencing has been completed and all the data for the three flow cells have been aligned, the alignment files (BAM format) from all the batches are combined, split into contig-wise alignment file using samtools^35^ and uploaded to the cloud storage bucket.

### Small-variant calling

#### Haplotype-aware variant calling pipeline

We used the PEPPER–Margin–DeepVariant^11^ pipeline to identify small-variants. The pipeline employs three modules: PEPPER, Margin and DeepVariant which together build a haplotype-aware variant caller and reports state-of-the-art, nanopore-based variant identification results^11,19^. An overview of this pipeline is presented here:

- **PEPPER SNP:** this finds SNPs from a read-to-reference alignment file using a recurrent neural network. First, PEPPER SNP generates nucleotide summary information for each position of the genome. Then the recurrent neural network takes the summary information as input and provides likelihood for observing alternative alleles at each position. Finally, the module reports potential SNP sites using the likelihood of observing alternative alleles^11^.
- **Margin:** this is a hidden Markov model (HMM)-based, haplotyping module that produces a haplotag for each read using the SNPs reported by PEPPER SNP. First, Margin extracts read substrings around SNP sites and generates alignment likelihoods between reads and alleles. Then it constructs an HMM to describe genotype and read bipartitions at each SNP site, enforcing consistent partitioning between sites. Margin runs the forward–backward algorithm on the HMM, and then marginalizes over all genotypes to find the most likely assignment of alleles to haplotypes. Finally, Margin uses maximum likelihood estimation to determine which haplotype best matches the read and assigns the read a haplotag. If a read spans no variants or has equal likelihood between haplotypes, then the read gets no haplotag.
- **PEPPER HP:** this takes the haplotagged alignment file from Margin and produces a set of candidate variants. First, PEPPER HP generates nucleotide summary information at each position of the genome for each haplotype. Then, a recurrent neural network takes the input from each haplotype to produce the likelihood of observing a base at each location. Finally, PEPPER HP reports a set of candidate variants using the haplotype-specific allele likelihoods.
- **DeepVariant:** this produces the final genotype calls by using a convolutional neural network with the candidates from PEPPER HP. DeepVariant represents sequence data as a pile-up of reads spanning a 221-bp region of the genome, with features of sequence data as different channels and (six) bases differing from the reference. Each candidate is classified as a homozygous reference, or a heterozygous or homozygous variant, with the probability of each state determining the genotype quality of the variant.

We accelerated the PEPPER–Margin–DeepVariant by running the pipeline independently on each contig of the reference genome that allowed use of 14 GPU-enabled compute instances on Google Cloud Platform (Supplementary Table 5). Once alignment has been completed, each variant calling instance downloads the alignment files for the contig(s) for which it is configured, combines it into a single contig-wise alignment file, performs variant calling and finally uploads the output (VCF file) to a Google Cloud storage bucket (Fig. 1b). Once all the runs have been completed, we downloaded all contig-wise VCF files in one instance and merged them to have variant calls for the whole genome.

To speed the subsequent small-variant filtration and prioritization, variants were overlapped with the ‘AllHomopolymers_gt6bp_imperfectgt10bp_slop5’ regions^36^ of the GIAB, annotated ‘Homopolymer’. Variants overlapping 4- to 6-bp homopolymer regions were annotated ‘ShortHomopolymer’. As VCF represents insertions at the previous base, annotation regions were extended 1 bp to the left. Negative scoring was also applied for homopolymer indel variants, because these variant types are the most redundant errors that we observed in ONT-based variant identification.

### SV calling and annotation

#### SV calling

For SV calling we used Sniffles^18^. Sniffles scans each of the reads for split events and alignment events (cigar). The latter most often indicates short SVs (50–500 bp) whereas split reads indicate larger SVs, especially rearrangements. Subsequently, Sniffles clusters the individual read information together, based on the break-point locations per SV and per read.

#### SV selection and tier grouping

After the calling, we selected rare SVs by comparing the SV calls with public and in-house catalogs of SVs derived from both short-read and long-read sequencing studies. We used the sveval package^37^ to match SV calls with SVs in these databases. Briefly, SVs are matched if the reciprocal overlap is >50% for deletions, inversions or duplications, or if the size is 50% similar for insertions located at <100 bp from each other. We removed SVs that matched an SV in the gnomAD-SV with an allele frequency >1%. We also called SVs using Sniffles across 11 genomes sequenced using Oxford Nanopore^9^. Again, we filtered SVs with an allele frequency >1% in this in-house catalog. Once rare SVs had been selected, we created three tiers of variants. Using Gencode v.35 gene annotation, we placed coding SVs in tier 1 and SVs in UTRs, promoters or introns of protein-coding regions in tier 2. Finally, noncoding SVs overlapping conserved regions and regulatory elements were placed in the third tier. The conserved region annotation was the *phastConsElements100way.txt.gz* track downloaded from the UCSC Genome Browser. As predicted regulatory elements, we used DNase hypersensitivity sites (*ENCFF509DLH*) and CTCF-binding site (*ENCFF415WKV*) tracks downloaded from the ENCODE portal^38,39^. In each tier, SVs associated with a gene from the prioritized gene list was highlighted first. The SVs were also compared with the Database of Genomic Variants^40^ and known clinical SVs in dbVar^41^ (study *nstd102*).

#### SV fine-tuning using local assembly

SVs in tier 1 (that is, coding SVs) were also fine-tuned using local assembly because base-pair resolution is particularly relevant to interpret variants potentially creating frameshifts. Reads supporting the SV, reported by Sniffles, were assembled into contigs using the Shasta assembler^9^. The assembled contigs were then aligned to the reference with minimap2 (ref. ^17^) and the SVs called using svim-asm^42^.

#### Read coverage track

In addition to Sniffles, we deployed mosdepth^43^ to compute the coverage per genome in 10,000-bp windows. This read coverage metrics served as orthogonal evidence for large deletions or duplications. We also used this information to investigate potential aneuploidy.

The SV annotation pipeline, including the local assembly module and read coverage module, is available at https://github.com/jmonlong/sv-nicu.

#### SV-calling benchmark

The accuracy of the SV calling was measured on the HG002 sample using the GIAB benchmark^15^. We used two evaluation methods to estimate the precision, recall and *F*1 score on the high-confidence regions provided by the GIAB: *truvari* (https://github.com/spiralgenetics/truvari) and *sveval* (https://github.com/jmonlong/sveval). We report the performance overall for each SV type. We note that the SV calls were more accurate for deletions (Supplementary Fig. 2).

#### Local assembly benchmark

We further used the GIAB benchmark to test the gain in base-pair resolution provided by our local assembly of the SVs in tier 1 (coding SVs). In the present study, we extracted all coding SVs in HG002 (that is, irrespective of their allele frequency) and ran the local assembly pipeline (above). The original SV calls and assembled calls were matched with the GIAB truthset using the *sveval* package^37^ as above. We then computed how many original SV calls and assembled SV calls matched the GIAB exactly, that is, having exactly the same positions and sizes. Out of the 56 deletions and 16 insertions in coding regions called by Sniffles, 23 deletions (41.1%) and 1 insertion (6.25%) matched exactly the SV in GIAB. After running our assembly pipeline, these numbers increased to 28 deletions (50%) and 7 insertions (43.8%). Hence, assembly of the SVs helps fine-tune the base-pair resolution of SV called by Sniffles, especially for insertions.

### Variant filtration and prioritization

#### Small-variant

The customized Alissa classification tree, including filtration and variant scoring, was developed against 15 clinical exome samples from our in-house genomics laboratory, the Stanford Clinical Genomics Program (CGP). The variants reported for these samples, 25 in total, included a wide variety of variant types, modes of inheritance and ACMG classifications of variant of uncertain significance (VUS), likely pathogenic or pathogenic. After iterative development and testing against these initial samples, the classification tree was validated against a separate set of clinical exome samples from CGP. As the objective of this analysis was to quickly flag clinically actionable, clearly pathogenic or likely pathogenic variants, a total of 15 exome samples with clinically positive reports of rare (minor allele frequency <1.0%) pathogenic or likely pathogenic variants (19 in total) were selected for validation. For the purposes of both development and validation, all samples were analyzed as proband cases, without gene target lists, even when the original clinical analyses included additional family members and/or gene target lists.

Iterative development against the initial test set of 15 samples ultimately produced a prioritization filtration scheme that assigned scores as follows: on average, 12 variants (range 6–16) per case received a score of ≥5; this included 12 of 15 of the reported pathogenic/likely pathogenic variants and 0 of 10 manually classified VUSs. On average, 26 variants (range 18–38) per case received a score of ≥4; this included 14 of 15 reported pathogenic/likely pathogenic variants and 3 of 10 manually classified VUSs. On average, 65 variants (range 51–85) per case received a score of ≥3; this included 15 of 15 of the reported pathogenic/likely pathogenic variants and 6 of 10 VUSs. The single likely pathogenic variant with a score of 3 was a missense change that has not been previously reported in the literature and is not predicted to be deleterious by in silico methods; our standard workflow was able to identify this variant only because the sample was originally analyzed as part of a trio, and this variant was identified as de novo and ultimately reported with partial clinical overlap. Based on these results, we chose a score of ≥4 as the cut-off most likely to flag pathogenic/likely pathogenic variants while still limiting the total pool of reviewed variants to a size compatible with a rapid time frame.

When validated against an additional 15 clinical exome samples, this rapid prioritization filtration scheme successfully identified 19 of 19 of the test variants (100%) with a score of ≥4, 16 of 19 (84%) with a score of ≥5 and 5 of 19 (26%) as the highest scoring variant in that analysis. Although possible values for scores ranged from 1 to 14, the highest score observed in this validation was 7.

Based on the performance of the customized Alissa classification tree with clinical exome data, our expectation was that application of this scheme to rapid genomic data would elevate pathogenic variants to a score of ≥4. The inclusion of target gene lists within the classification tree would be to increase scores of clinically significant variants (variable points can be awarded per case, based on the confidence of the patient-specific target list).

It should be noted that, although both systems utilize patient-specific target gene lists, neither system limits analysis to that list. However, the two systems are implemented in different ways: in the standard system, variants are limited to protein-coding variants before evaluation of the target gene lists, whereas in the rapid system the scores are assigned for the target gene list before evaluation of coding impact. As a result, the analysis included a number of variants that were scored as being on the target list, but that had no significant protein impact (for example, synonymous variants outside the intron–exon junction).

We also examined the differences between the standard and accelerated pipelines for the patient samples in more detail; variants were divided into ordered, exclusive groups based on the criterion that first triggered manual review in the standard system: variants that (1) have pathogenic or likely pathogenic classifications in ClinVar (Supplementary Table 16); (2) have HGMD^30^ disease-causing mutation (DM) or DM? annotations (Supplementary Table 17); (3) are on the target list (Supplementary Table 18); (4) have recessive inheritance (Supplementary Table 19); and (5) have predicted deleterious protein impact (Supplementary Table 20). Each variant marked for review by the rapid system was also reviewed to determine the criteria that contributed to a final score of ≥4. Categories (1) (ClinVar annotations), (2) (HGMD annotations) and (4) (recessive inheritance) showed the anticipated result: the standard and rapid systems identified similar numbers of variants as candidates, but the rapid system marked only a subset for review. Categories (3) (target gene variants) and 5 (predicted deleterious protein impact) had a different result, in that the rapid system scored a larger number of variants than the standard system, but then marked a similar number for review.

#### Structural variation

Copy number and SVs were prioritized when they overlapped coding regions of genes or any region of a gene on the target panel. Regions were excluded from further consideration when concordant variants in the Database of Genomic Variants were identified. Genes that contained variants not excluded due to population variation were then examined for potential clinical overlap with patient’s reported features using OMIM, PubMed and HGMD.

### Analysis of clinically relevant variants

Alissa filters were used to identify the annotated pathogenic or likely pathogenic variants in each sample. The QIAGEN HGMD Professional filter identified variants annotated as disease-causing mutations in the HGMD (QIAGEN HGMD Professional Database 2020.4). This filter mapped variants to HGMD based on genomic location, and those annotated with either high (DM) or low (DM?) confidence were treated as pathogenic/likely pathogenic.

The candidate ‘clinical’ variants were generated by passing all the variants from a sample through the Alissa filter, without any additional previous filtering. The benchmark consisted of the filtered GIAB HG002 v.4.2.1 variants. The variants generated from our pipeline for the nonbarcoded HG002 sample were compared with the PrecisionFDA Illumina sample variants (https://storage.googleapis.com/brain-genomics-public/research/sequencing/grch37/vcf/novaseq/wgs_pcr_free/30x/HG002.novaseq.pcr-free.30x.deepvariant-v1.0.grch37.vcf.gz). The analysis is provided in Supplementary Table 13.

### Hardware infrastructure

The stage-wise hardware requirements for the ultra-rapid pipeline, as implemented on the Google Cloud Platform, have been presented in Supplementary Table 21. The pipeline can be used with similar configurations on other cloud platforms. For base calling, our system requires a total of 64 NVIDIA V100 GPUs (the GPUs are mainly chosen based on the Guppy base calling implementation available; they might move to a different GPU type in the future; we used the state-of-the-art hardware). The hardware requirement is based on keeping up with the sequencing time for fresh flow cells. As the flow cells are reused, the rate of throughput decreases, which in turn reduces the compute requirement, specifically the number of GPUs for base calling. For small-variant calling, the system required a higher CPU:GPU ratio and, based on the configurations available in the cloud platform, we chose the NVIDIA P100 GPUs. Ideally, for local implementation, we would require a system with 64 NVIDIA V100 and around 96 CPU cores per GPU and NVME-based storage (which is very common in modern systems). With such a cluster (the node-wise configuration does not matter particularly, because our system allows for specifying the node-wise resource configuration), we use the same hardware for the near real-time base calling and alignment and fast small-variant calling. The hardware requirement for Sniffles is low and requires only 192 CPU cores. In terms of software, our system can easily be changed to accommodate a local deployment.

### Reporting Summary

Further information on research design is available in the Nature Research Reporting Summary linked to this article.

## Online content

Any methods, additional references, Nature Research reporting summaries, source data, supplementary information, acknowledgements, peer review information; details of author contributions and competing interests; and statements of data and code availability are available at 10.1038/s41587-022-01221-5.

## Supplementary information

Supplementary Information Supplementary Notes, Figs. 1–4, and Tables 1–21.

Reporting Summary

## Data Availability

We have made the data for the HG002 sample we sequenced available at the following links (fastq files): https://storage.googleapis.com/ur_wgs_public_data: (1) barcoded sample: *HG002_BC04.fastq.gz;* (2) nonbarcoded sample: HG002_No_BC.fastq.gz. The nonbarcoded sample data were also used for the runtime and accuracy analysis presented in Results. We used the publicly available GRCh37 human genome reference: https://ftp-trace.ncbi.nih.gov/1000genomes/ftp/technical/reference/phase2_reference_assembly_sequence/hs37d5.fa.gz. The BED files with the regions for the small-variant call annotation is available at https://storage.googleapis.com/ur_wgs_public_data/small_variant_annotation. (1) ‘Homopolymer’: *GRCh37_AllHomopolymers_gt6bp_imperfectgt10bp_slop5.bed.gz;* (2) ‘ShortHomopolymer’: *grch37.4bp_to_6bp_homopolymers_left_pad_1bp.bed.* The gene lists used for the patient samples are available at: https://storage.googleapis.com/ur_wgs_public_data. We used the following publicly available databases for variant filtration and prioritization: (1) NCBI ClinVar 2020-12; (2) OMIM 2021-01-06; (3) gnomAD release 2.0.2; (4) RefSeq Transcripts v.91 released 9 November 2018 (accession no. NM_001001430.1 for patient 1). We also used the commercially available QIAGEN HGMD Professional Database 2020.4. The data for the patient samples cannot be shared under the restrictions placed by the institutional review board. Source data are provided with this paper.

## Source data

Source Data Fig. 2 Statistical data.

## Code availability

The scripts for running the pipeline along with the detailed instructions on running the test data are provided at https://github.com/gsneha26/urWGS.
