# Supplementary material for: Accelerated identification of disease-causing variants with ultra-rapid nanopore genome sequencing
Source: Nat Biotechnol. 2022 Mar 28;40(7):1035–41. doi: 10.1038/s41587-022-01221-5 (PMC9287171; doi:10.1038/s41587-022-01221-5)
Supplement: Supplementary file 1 — Supplementary Notes, Figs. 1–4, and Tables 1–21. [file 41587_2022_1221_MOESM1_ESM.pdf]

nature portfolio

https://doi.org/10.1038/s41587-022-01221-5

Supplementary information

Accelerated identification of disease-causing variants with ultra-rapid nanopore genome sequencing

In the format provided by the authors and unedited

Supplementary Notes

Sequencing summary analysis

We concatenated the sequencing summary generated from Guppy across all the base calling jobs across a sample and extracted summary statistics with NanoStat using the following command:

```
python3 NanoStat.py --summary SEQUENCING_SUMMARY_FILE.txt > SUMMARY_STATISTICS.txt
```

An additional

```
--barcoded
```

option was added when the barcoded HG002 sample was analysed.

Read carryover experiment

We extracted a fraction of reads from HG005 using samtools with the following command:

```
samtools view -b -s 0.FRAC \
    -@<THREADS> \
    -b HG005_INPUT.bam > HG005_CARRYOVER.bam
```

And merged the reads from HG005 to HG002 using the following command:

```
samtools merge -@71 out.bam HG002_ALIGNMENT.bam HG005_CARRYOVER.bam
```

Note that our pipeline expects a single sample bam file, so there was no use of read groups or sample name in the alignment files.

Stratified variant accuracies

We used `hap.py` version `v0.3.12` to assess the variant calls against GIAB truth set. The `hap.py` program is available via `jmcdani20/hap.py:v0.3.12` docker image.

```
docker run -it jmcdani20/hap.py:v0.3.12 /opt/hap.py/bin/hap.py \
    HG002_GRCh37_1_22_v4.2.1_benchmark.vcf.gz \
    VARIANTS.vcf \
    -f HG002_GRCh37_1_22_v4.2.1_benchmark_noinconsistent.bed \
    -r hs37d5.fa \
    --stratification=STRATIFICATION.tsv
    -o output/ --pass-only --no-roc --no-json --engine=vcfeval --threads=32
```

Plots were generated using the script `plot_stratified_happy_results.py` in the repository at:
`https://github.com/tpesout/genomics_scripts/tree/2d2a4bbb28a8c77ca7c15f771638d919e9d03cc1`

Genome-In-A-Bottle (GIAB) benchmarking data was acquired from:
ftp://ftp-trace.ncbi.nlm.nih.gov/giab/ftp/release/AshkenazimTrio/HG002_NA24385_son/NISTv4.2.1/GRCh37/

Gencode gene regions (for stratification) was found here:
ftp://ftp.ebi.ac.uk/pub/databases/gencode/Gencode_human/release_35/gencode.v35.annotation.gtf.gz

Other genomic stratification regions were found here:
https://ftp-trace.ncbi.nlm.nih.gov/giab/ftp/release/genome-stratifications/v2.0/

Variant Statistics

To determine Transistion/Transversion (Ti/Tv) and Heterozygous/Homozygous (Het/Hom) statistics on our variants described in Supplementary Table 11, we used Real Time Genomics's (https://github.com/RealTimeGenomics/rtg-tools) `vcfstats` command:

```
rtg vcfstats VARIANTS.vcf
```

Further Disclosures

Google employees did not have access to patient data.

Supplementary Figures

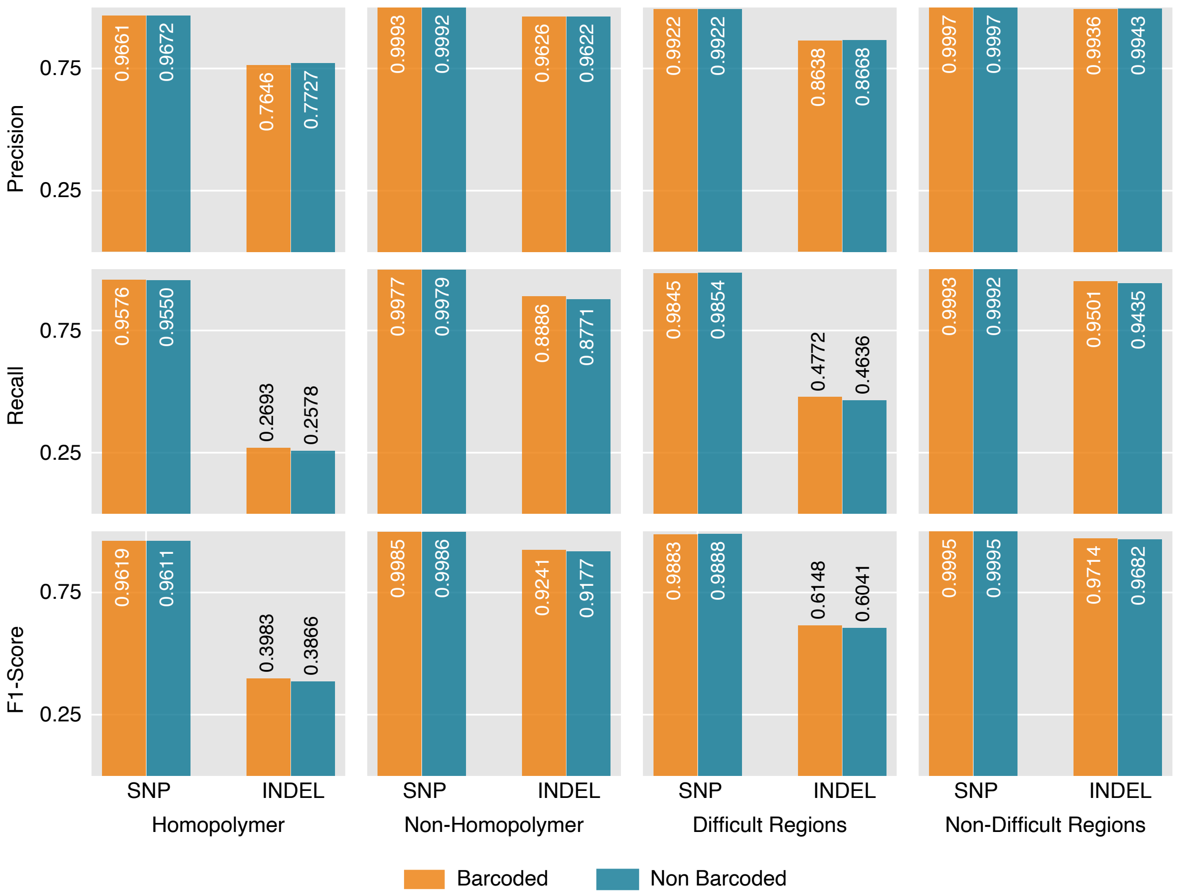

Supplementary Figure S1: Stratified variant calling performance comparison between barcoded and non-barcoded HG002 sample in homopolymer, non-homopolymer difficult and non-difficult regions. The HG002 non-barcoded run was the 7th sample run on the flow cells. The similarity in variant calling performance shows that barcoding is not necessary to achieve high-quality variant calls.

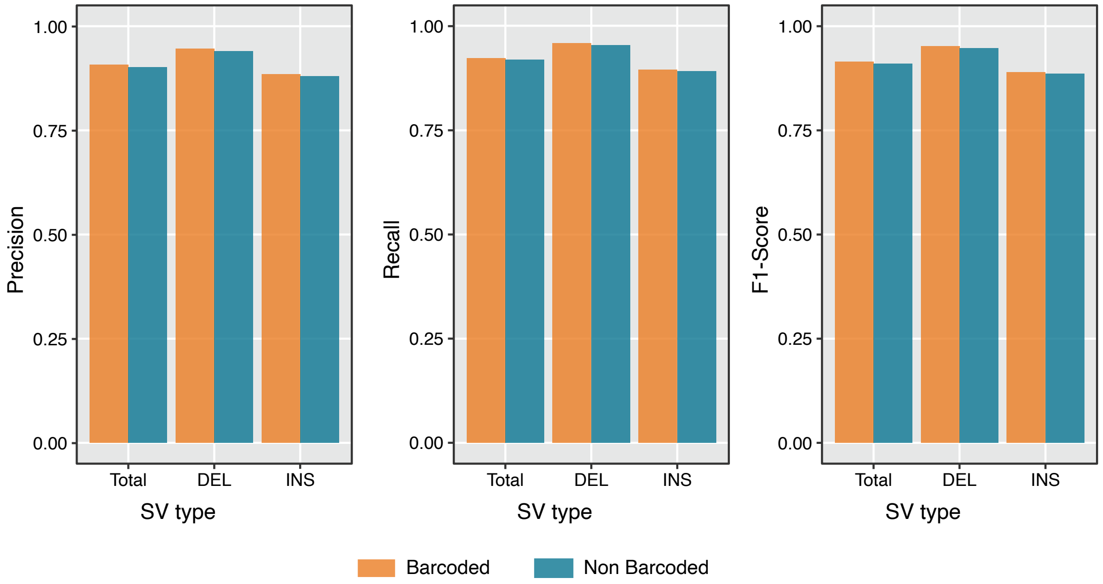

Supplementary Figure S2: Structural variant (SV) calls from Sniffles were compared with the GIAB truthset for the HG002 sample. The bars show the F1 score (left panel), precision (center panel), and recall (right panel) computed by truvari. The benchmark focused on SVs called in the high-confidence regions provided by the GIAB benchmark. Both the barcoded and non-barcoded samples (colors) show similar accuracy.

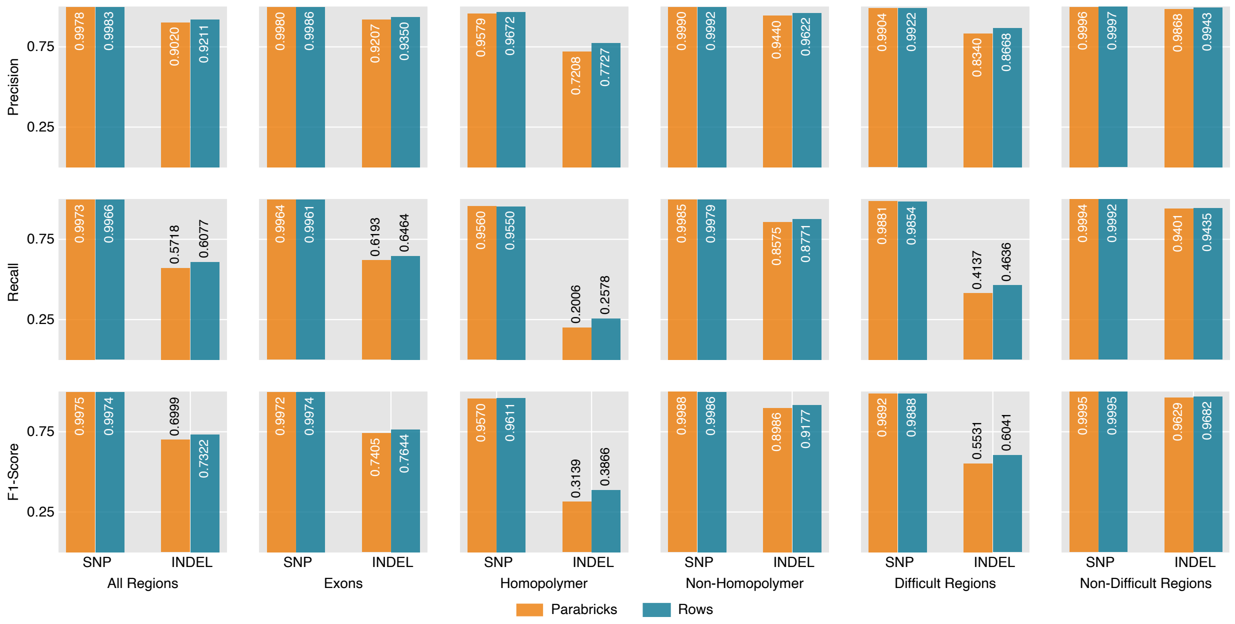

Supplementary Figure S3: Stratified variant calling performance comparison between Parabricks DeepVariant and Google DeepVariant with rows model. An improvement with the rows model, especially in the INDEL F1-score is seen across the different regions.

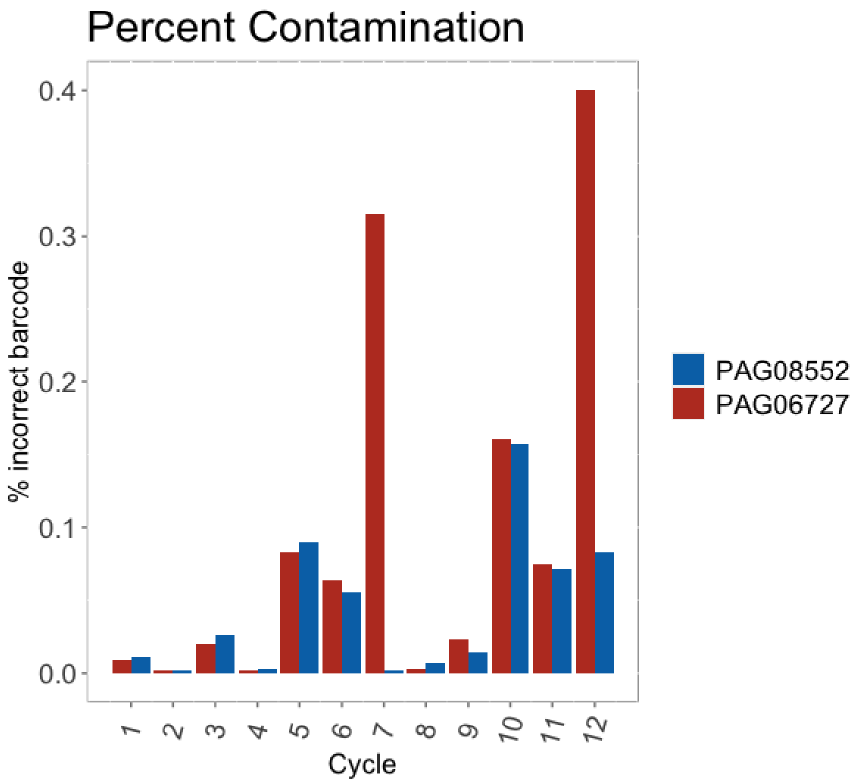

Supplementary Figure S4: Percent carryover over 12 runs, each one following a run and change in barcode of the sample. carryover rate was measured independently over two flow cells (PAG08552-Blue, and PAG06727-Red).

Supplementary Tables

**Kit**	**Total DNA recovered from 1.6ul blood (ug)**	**Sample Purity**	**Mean Fragment Length**	**Time (min)**
**Puregene**	36	1.7	>60kb	50
**DNeasy**	16	1.8	23kb	27

Supplementary Table 1: Comparison of the time for DNA extraction with 2 different kits - Puregene and DNeasy accompanied with the total DNA recovered, sample purity and the mean fragment length. We chose Puregene kit since it has more than double DNA recovery from the same volume of blood sample with a much higher mean fragment length.

	HG002 chr20 no carryover		HG002 chr20 1% carryover with HG005	
**Type**	INDEL	SNP	INDEL	SNP
**TRUTH.TOTAL**	42689	264143	42689	264143
**TRUTH.TP**	23216	263238	23151	263149
**TRUTH.FN**	19473	905	19538	994
**QUERY.TOTAL**	40068	366375	39966	366543
**QUERY.FP**	3743	1468	3737	1513
**FP.gt**	1276	160	1320	231
**Recall**	0.54384	0.996574	0.542318	0.996237
**Precision**	0.86397	0.994456	0.863906	0.994285
**F1-Score**	0.667507	0.995514	0.66634	0.99526

Supplementary Table 2: Results of the in-silico carryover experiment on HG002 chr20 sample. The carryover was modeled by adding 1% reads from HG005 (different ethnicity). The variant call performance for both the samples - with 1% carryover and no-carryover - are evaluated using the GIAB benchmarks for HG002. The differences are negligible and demonstrate that our variant calling pipeline is robust to at least 1% carryover. Details of the metrics are provided in https://github.com/Illumina/hap.py/blob/master/doc/happy.md#full-list-of-output-columns

	**HG002 (Barcoded)**	**HG002 (Not Barcoded)**
**Reads (total)**	23,136,167	28,976,978
**Reads (Passed)**	19,883,827	24,578,016
**Reads (Passed %)**	85.94	84.8
**Reads (Proper Barcode)**	19,512,448	N/A
**Reads (Passed with Proper Barcode)**	18,721,976	N/A
**N50**	20427	18818
**Bases (total (Gb))**	209.97	219.85
**Bases (Passed (Gb))**	186.55	192.24
**Bases (Passed %)**	88.85	87.4
**Bases (Proper Barcode (Gb))**	184.33	N/A
**Bases (Passed with Proper Barcode (Gb))**	177.42	N/A

Supplementary Table 3: Sequencing summary statistics for the barcoded and the non-barcoded HG002 sample. Reads are categorized as "pass" by Guppy if the corresponding strand q-score ≥ 7.

	HG002 (Barcoded)		HG002 (Not Barcoded)	
**Type**	INDEL	SNP	INDEL	SNP
**TRUTH.TOTAL**	522391	3352686	522391	3352686
**TRUTH.TP**	323479	3341149	317464	3341409
**TRUTH.FN**	198912	11537	204927	11277
**QUERY.TOTAL**	479097	3807794	465123	3807293
**QUERY.FP**	29247	5528	27581	5550
**FP.gt**	14849	1240	14543	1320
**Recall**	0.619228	0.996559	0.607713	0.996636
**Precision**	0.918227	0.998349	0.921111	0.998342
**F1-Score**	0.739653	0.997453	0.73229	0.997489

Supplementary Table 4: The variant call performance for HG002 barcoded and non-barcoded samples are evaluated using the GIAB benchmarks for HG002. Details of the metrics are provided in https://github.com/Illumina/hap.py/blob/master/doc/happy.md#full-list-of-output-columns. The differences are negligible.

	**Female**		**Male**	
**Instance**	**Contig1**	**Contig2**	**Contig1**	**Contig2**
**1**	chr1		chr1	
**2**	chr2		chr2	
**3**	chr3		chr3	
**4**	chr4	chrMT	chr4	chrMT
**5**	chr5	chrY	chr5	chr22
**6**	chr6	chr22	chr6	chrY
**7**	chr7	chr19	chr7	chr18
**8**	chr8	chr20	chr8	chr19
**9**	chr9	chr13	chr9	chrX
**10**	chr10	chr18	chr10	chr15
**11**	chr11	chr14	chr11	chr20
**12**	chr12	chr15	chr12	chr14
**13**	chrX	chr21	chr13	chr17
**14**	chr16	chr17	chr16	chr21

Supplementary Table 5: Contig distribution across the small variant calling instances based on the gender of the patient

	Google (none model) Runtime (min)			Parabricks Runtime (min)			Google (rows model) Runtime (min)		
**Instance**	**Contig1**	**Contig2**	**Total**	**Contig1**	**Contig2**	**Total**	**Contig1**	**Contig2**	**Total**
**1**	36		36	20		20	45		45
**2**	37		37	21		21	45		45
**3**	31		31	17		17	38		38
**4**	31	3	34	17	2	19	39	2	41
**5**	29	8	37	16	5	21	36	10	46
**6**	28	10	38	16	7	23	35	11	46
**7**	16	12	28	14	8	22	32	13	45
**8**	27	11	38	14	7	21	33	14	47
**9**	22	14	36	12	9	21	27	16	43
**10**	22	14	36	13	8	21	28	17	45
**11**	22	11	33	12	7	19	27	17	44
**12**	22	15	37	12	9	21	28	19	47
**13**	18	20	40	10	13	23	25	22	47
**14**	20	9	29	13	5	18	24	11	35
**Actual Runtime**			**40**			**23**			**47**
**Sequential Runtime**			**488**			**282**			**610**
**Speedup**			**12.25x**			**12.5x**			**13x**

Supplementary Table 6: Comparison of small variant calling contig-wise runtime for default Google DeepVariant vs Parabricks DeepVariant vs Google DeepVariant with rows model for the HG002 sample.

	Instance 1 contigs	Runtime (min)	Instance 2 contigs	Runtime (min)
	chr1	19	chr16	29
	chr2	19	chr4	15
	chr3	16	chr5	15
	chr6	14	chr7	14
	chr12	12	chr8	12
	chr13	9	chr10	11
	chr9	11	chr11	11
	chr17	9	chr14	8
	chrX	7	chr15	7
	chr22	4	chr18	7
	chrY	2	chr19	5
	chrMT	1	chr20	6
			chr21	4
**Actual Runtime**		**19**		**29**

Supplementary Table 7: Contig distribution and runtime across the structural variant calling instances

	Sample	HG002	HG002	Patient 2
	**Pipeline**	Parabricks DV	Google DV (rows)	Google DV (rows)
	**Exome**	60,382	59,772	61,398
	**Mapped Variants**	69,532	68,835	70,881
	**MAF<0.5%**	8,363	7,809	9,369
	**target list**	25	24	32
**off target list**	**total**	8,338	7,785	9,337
	**off target: coding**	2,151	1,927	2,534
	**off target: non-coding or synonymous**	6,187	5,858	6,803
	**not disease annotated**	1,952	1,755	2,304
	**OMIM morbid**	221	193	256
**Score >0**	**total**	167	147	209
	**1**	72	62	99
	**2**	48	46	49
	**3**	27	21	30
	**4**	12	12	20
	**5**	4	3	3
	**6**	3	2	7
	**7**	1	1	0
	**8**	0	0	0
	**9**	0	0	0
	**10**	0	0	0
	**11**	0	0	0
	**12**	0	0	1
	**Score ≥ 4 (Reviewed)**	20	18	31
	**Variants marked for review in standard workflow**	101	101	147

Supplementary Table 8: Number of small variants available at every stage of the filtration and prioritization process for 3 cases - HG002 sample with Parabricks DeepVariant pipeline, HG002 sample with Google DeepVariant and rows model pipeline, patient sample with Google DeepVariant and rows model pipeline. All the variants in the target list are considered for prioritization. In case of variants off the target list, only the protein coding variants are evaluated. The number of variants marked for manual review with the standard pipeline has also been presented. The number of variants is almost $5\times$ less with the ultra-rapid pipeline as compared to the standard pipeline.

**Rarity**	**Prioritization Data Category**	**Criteria**
MAF<5.0%	Classifications in ClinVar	Pathogenic or Likely Pathogenic
MAF<0.5%	HGMD disease association	DM or DM?
	Target Gene List	Nonsense, Frameshift, Start or stop loss
		Expanded splice junction<br>(+/- 10 bp intronic & +/- 2 bp exonic)
		Missense
		In-frame indels
	Recessive	Associated with any disease in OMIM
	Protein Impact	Nonsense, Frameshift,<br>start or stop loss
		Cannonical splice junction
		Missense and consensus deleterious prediction<br>(MutationTaster = Disease Causing &<br>Missense & RVIS <25% &<br>gnomAD Missense z score >2 &<br>CAD score >= 20)

Supplementary Table 9: standard Filtration Scheme: triggers for review all variants meeting a single criterion.

Rarity	Prioritization Data Category	Prioritization Data	Points
MAF<0.5%	Classifications in ClinVar	Pathogenic or Likely Pathogenic	2
		Benign or likely Benign (without Pathogenic/likely Pathogenic)	-1
	HGMD disease association	High Confidence (DM)	2
		Low Confidence (DM?)	1
	Target Gene List	Any	2
		Expanded splice junction (+/- 10 bp intronic & +/- 2 bp exonic)	2
		LoF & pLi>0.9 or o/e>0.35	1
		Missense or in-frame indel	1
	Recessive (gene has at least two variants)	Phased biallelic	1
		Associated with recessive disease in OMIM	1
	Dominant (gene has only one variant)	Associated with dominant disease in OMIM	1
	Protein Impact	Nonsense, Frameshift, start or stop loss	3
		Cannonical splice junction	3
		LoF & pLi>0.9 or o/e>0.35	1
		Missense & MutationTaster = Disease Causing	1
		Missense & RVIS <25%	1
		Missense & gnomAD Missense z score >2	1
	Quality	Variant within a homopolymer region	-2

Supplementary Table 10: Ultra-Rapid Filtration Scheme: variants with total scores of 4 or greater were manually reviewed

	SNP	INDEL	Ti/Tv Ratio	Het/Hom Ratio
**Patient 1**	3834894	481570	2.02	1.29
**Patient 2**	3992032	489770	2.02	1.58

Supplementary Table 11: Small variant calling statistics for the patient samples – number of SNPs and INDELs, Transition/Tranversion ratio and Heterozygous/Homozygous ratio

Type	Subset	Recall	Precision	F1-Score	TP	FN	FP
INDEL	all regions	0.997562	0.992654	0.995102	518817	1268	4001
	CDS	0.992337	0.992381	0.992359	518	4	4
	exons	0.996991	0.992566	0.994774	17230	52	133
	genes	0.997575	0.99281	0.995187	315987	768	2383
SNP	all regions	0.998776	0.994457	0.996612	3334665	4086	18592
	CDS	0.997024	0.99108	0.994043	22110	66	199
	exons	0.997712	0.99342	0.995562	140874	323	933
	genes	0.998777	0.994754	0.996762	1977718	2421	10431

Supplementary Table 12: Small variant calling statistics for Illumina-based variant calling of HG002 against GIAB v4.2.1 variants on GRCh37 stratified across gene regions annotated by GENCODE v35.

Sample	type	TP	FN	FP	Precision	Recall
**Illumina**	**SNP**	189	0	11	0.945	1.0
	**INDEL**	24	1	10	0.706	0.96
**Oxford Nanopore**	**SNP**	188	1	11	0.945	0.995
	**INDEL**	17	8	7	0.708	0.68

Supplementary Table 13: Small variant calling statistics for the clinically relevant variants in the non-barcoded HG002 sample and the Illumina HG002 sample.

Flow cell	Cycle	%passed reads	%unclassified reads	%incorrect reads
**PAG06727**	1	95.82	9.16	0.008559541
	2	94.85	5.60	0.001391214
	3	95.48	16.54	0.020177902
	4	95.59	13.65	0.002130515
	5	94.59	7.48	0.082866044
	6	94.82	7.29	0.063324568
	7	94.26	8.04	0.31511914
	8	94.00	6.49	0.002754702
	9	93.42	11.93	0.023506629
	10	93.65	21.32	0.160611359
	11	93.23	11.30	0.07499163
	12	92.20	14.18	0.40062083
**PAG08552**	1	94.08	8.80	0.010601009
	2	93.33	5.54	0.001527824
	3	95.34	16.28	0.02616143
	4	94.83	13.28	0.002877919
	5	93.20	7.70	0.089918069
	6	90.95	7.31	0.055346697
	7	92.63	8.61	0.001757624
	8	89.91	6.53	0.007202795
	9	90.03	12.65	0.013730671
	10	91.47	23.32	0.157410487
	11	88.95	12.11	0.071325688
	12	87.84	12.87	0.082960702

Supplementary Table 14: Read Statistics for the carryover experiment across the 12 cycles and 2 flow cells. Failed (Q score < 7) and unclassified reads are discarded. % incorrect reads/carryover rate is calculated as the % of passed, classified reads that do not correspond to the correct barcode. Highest carryover of 0.4% is observed.

Instance Number	Flow cells	Time (hr)	Total Throughput (Gb)
**1**	1A, 2A, 3A	1.9	14.59
**2**	4A, 5A, 6A	1.9	13.64
**3**	1B, 2B, 3B	1.7	12.04
**4**	4B, 5B, 6B	1.9	14.33
**5**	1C, 2C, 3C	1.9	15.16
**6**	4C, 5C, 6C	1.9	14.9
**7**	1D, 2D, 3D	1.9	14.72
**8**	4D, 5D, 6D	1.9	14.24
**9**	1E, 2E, 3E	1.9	14.85
**10**	4E, 5E, 6E	1.7	12.65
**11**	1F, 2F, 3F	1.75	13.71
**12**	4F, 5F, 6F	1.7	11.98
**13**	1G, 2G, 3G	1.9	13.45
**14**	4G, 5G, 6G	1.9	14.85
**15**	1H, 2H, 3H	1.7	11.86
**16**	4H, 5H, 6H	1.75	12.85

Supplementary Table 15: Flow cell distribution across the base calling and alignment compute instances for a total sequencing time of 90 minutes. While the throughput ranges between 11.86 Gb and 15.16 Gb, the difference in runtime is 12 minutes - resulting in a low tail latency differences among the instances.

		Prioritization data applied to variants reviewed in Ultra-Rapid Filtration Scheme					
**Variant ID**	**Total Score**	**ClinVar Pathogenic / Likely Pathogenic**	**HGMD Disease Mutation**	**Target Gene List**	**Loss-of-Function**	**Deleterious Missense**	**Dominant**
Var1	4	2	2	0	0	0	0
Var2	6	2	2	0	0	1	1
Var3	6	2	2	0	0	1	1
Var4	6	2	2	0	0	1	1
Var5	12	2	2	4	3	0	1

Supplementary Table 16: Score breakdown for variants that have Pathogenic or Likely Pathogenic classifications in ClinVar

		Prioritization data applied to variants reviewed in Ultra-Rapid Filtration Scheme						
**Variant ID**	**Total Score**	**HGMD Disease Mutation**	**Target Gene List**	**Loss-of-Function**	**Deleterious Missense**	**Dominant**	**Homopolymer**	**Benign/ Likely Benign**
Var6	4	2	0	3	0	1	-2	0
Var7	4	2	0	0	1	1	0	0
Var8	6	2	3	0	2	0	0	-1
Var9	6	1	3	0	1	1	0	0
Var10	4	1	0	0	2	1	0	0

Supplementary Table 17: Score breakdown for variants that have HGMD DM or DM? annotation, but not Pathogenic or Likely Pathogenic classifications in ClinVar

		Prioritization data applied to variants reviewed in Ultra-Rapid Filtration Scheme				
**Variant ID**	**Total Score**	**Target Gene List**	**Loss-of-Function**	**Deleterious Missense**	**Dominant**	**Benign/ Likely benign**
Var11	4	4	0	0	0	0
Var12	4	4	0	0	1	-1
Var13	4	3	0	1	0	0
Var14	4	3	0	1	0	0
Var15	4	3	0	1	0	0
Var16	4	3	0	1	0	0
Var17	5	3	0	1	1	0
Var18	5	3	0	2	0	0
Var19	6	3	0	2	1	0
Var20	6	3	0	2	1	0
Var21	5	2	3	0	0	0

Supplementary Table 18: Score breakdown for variants that are on the target list, but do not have HGMD DM or DM? annotation or Pathogenic or Likely Pathogenic classifications in ClinVar

		Prioritization data applied to variants reviewed in Ultra-Rapid Filtration Scheme	
**Variant ID**	**Total Score**	**Recessive**	**Loss-of-Function**
Var22	4	1	3
Var23	4	1	3
Var24	4	1	3
Var25	4	1	3
Var26	4	1	3

Supplementary Table 19: Score breakdown for variants that have potentially recessive inheritance, but are not on the target list, and do not have HGMD DM or DM? annotation or Pathogenic or Likely Pathogenic classifications in ClinVar

		Prioritization data applied to variants reviewed in Ultra-Rapid Filtration Scheme		
**Variant ID**	**Total Score**	**Dominant**	**Loss-of-Function**	**Deleterious Missense**
Var27	4	1	3	0
Var28	4	1	3	0
Var29	4	1	0	3
Var30	4	1	0	3
Var31	4	1	0	3

Supplementary Table 20: Score breakdown for variants that have deleterious protein impact, but are not recessive, are not on the target list, and do not have HGMD DM or DM? annotation or Pathogenic or Likely Pathogenic classifications in ClinVar

Stage	Instance Type	No. of Instances	GPUs/ Instance	virtual CPUs (vCPUs)	NVME-based local SSD
**Base calling, Alignment**	custom-48-204800	16	4× NVIDIA Tesla V100	48	3x375GB
**Small Variant calling**	n1-standard-96	14	4× NVIDIA Tesla P100	96	1x375GB
**Structural Variant calling**	n1-standard-96	2		96	1x375GB
**Variant Call Annotation**	n1-standard-96	1		96	1x375GB

Supplementary Table 21: Details about the instance configurations and number of instances for every stage in the pipeline - (a) Base calling, Alignment (b) Small Variant calling (c) Structural Variant Calling (d) Variant Call Annotation. Virtual CPUs in the instances refers to the hardware level implementation of multithread available on a CPU processor. Base calling with Guppy and Small Variant calling processes require GPUs. Small Variant calling, specifically PEPPER requires a higher vCPU to GPU ratio, so based on the GPU-based instance configuration constraints from Google Cloud, we chose the less powerful P100 GPU which came with 96 vCPUs. Due to the large number and/or size of files that are transferred between the instances and the storage bucket and the large number of file I/O operations, local SSD with NVME interface are used in all the instances.
