# Supplementary material for: Accelerated identification of disease-causing variants with ultra-rapid nanopore genome sequencing
Source: Nat Biotechnol. 2022 Mar 28;40(7):1035–41. doi: 10.1038/s41587-022-01221-5 (PMC9287171; doi:10.1038/s41587-022-01221-5)
Supplement: Supplementary file 2 — Reporting Summary [file 41587_2022_1221_MOESM2_ESM.pdf]

nature portfolio

Corresponding author(s): Euan A. Ashley

Last updated by author(s): Jan 11, 2022

Reporting Summary

Nature Portfolio wishes to improve the reproducibility of the work that we publish. This form provides structure for consistency and transparency in reporting. For further information on Nature Portfolio policies, see our Editorial Policies and the Editorial Policy Checklist.

Statistics

For all statistical analyses, confirm that the following items are present in the figure legend, table legend, main text, or Methods section.

n/a	Confirmed	
☐	☒	The exact sample size (*n*) for each experimental group/condition, given as a discrete number and unit of measurement
☒	☐	A statement on whether measurements were taken from distinct samples or whether the same sample was measured repeatedly
☒	☐	The statistical test(s) used AND whether they are one- or two-sided<br>*Only common tests should be described solely by name; describe more complex techniques in the Methods section.*
☒	☐	A description of all covariates tested
☒	☐	A description of any assumptions or corrections, such as tests of normality and adjustment for multiple comparisons
☐	☒	A full description of the statistical parameters including central tendency (e.g. means) or other basic estimates (e.g. regression coefficient) AND variation (e.g. standard deviation) or associated estimates of uncertainty (e.g. confidence intervals)
☒	☐	For null hypothesis testing, the test statistic (e.g. *F*, *t*, *r*) with confidence intervals, effect sizes, degrees of freedom and *P* value noted<br>*Give P values as exact values whenever suitable.*
☒	☐	For Bayesian analysis, information on the choice of priors and Markov chain Monte Carlo settings
☒	☐	For hierarchical and complex designs, identification of the appropriate level for tests and full reporting of outcomes
☒	☐	Estimates of effect sizes (e.g. Cohen's *d*, Pearson's *r*), indicating how they were calculated

Our web collection on statistics for biologists contains articles on many of the points above.

Software and code

Policy information about availability of computer code

Data collection	Data acquisition was done using Oxford Nanopore Technologies MinKnow software that comes with the PromethION 48 sequencing device by default.
Data analysis	urWGS: hhttps://github.com/gsneha26/urWGS (MIT license)<br>Guppy: Oxford Nanopore Technology provided basecaller v4.2.2 (commercial product)<br>Minimap2: https://github.com/lh3/minimap2 v2.17 (MIT License)<br>samtools: https://github.com/samtools/samtools v1.11 (MIT License)<br>PEPPER: https://github.com/kishwarshafin/pepper r0.5 (MIT License)<br>Margin: https://github.com/UCSC-nanopore-cgl/margin r0.5 (MIT License)<br>Google DeepVariant: https://github.com/google/deepvariant (BSD-3-Clause License)<br>Parabricks DeepVariant: Provided by NVIDIA v3.5 (commercial product)<br>Sniffles: https://github.com/fritzsedlazeck/Sniffles v1.0.12 (MIT License)<br>SV annotation: https://github.com/jmonlong/sv-nicu v0.5<br>NanoStat: https://github.com/wdecoster/nanostat (GPL-3.0 License)<br>bcftools: https://github.com/samtools/bcftools (MIT License)<br>hap.py: https://github.com/Illumina/hap.py v0.3.12 (BSD license)<br>vcf stratification analysis: https://github.com/tpesout/genomics_scripts (MIT License)<br>vcf statistics: https://github.com/RealTimeGenomics/rtg-tools (BSD-2-Clause License)<br>truvari: https://github.com/spiralgenetics/truvari (MIT License)<br>sveval: https://github.com/jmonlong/sveval (MIT License)

For manuscripts utilizing custom algorithms or software that are central to the research but not yet described in published literature, software must be made available to editors and reviewers. We strongly encourage code deposition in a community repository (e.g. GitHub). See the Nature Portfolio guidelines for submitting code & software for further information.

Data

Policy information about availability of data

All manuscripts must include a data availability statement. This statement should provide the following information, where applicable:

- Accession codes, unique identifiers, or web links for publicly available datasets
- A description of any restrictions on data availability
- For clinical datasets or third party data, please ensure that the statement adheres to our policy

We have made the data for the HG002 sample we sequenced, available at the following links (fastq files):
https://storage.googleapis.com/ur_wgs_public_data/
1. Barcoded sample: HG002_BC04.fastq.gz
2. Non-Barcoded sample: HG002_No_BC.fastq.gz
The non-barcoded sample data was also used for the runtime and accuracy analysis presented in the results section.

We used the publicly available GRCh37 human genome reference: ftp://ftp-trace.ncbi.nih.gov/1000genomes/ftp/technical/reference/phase2_reference_assembly_sequence/hs37d5.fa.gz.

The BED files with the regions for the small variant call annotation is available at https://storage.googleapis.com/ur_wgs_public_data/small_variant_annotation/
1. “Homopolymer”: GRCh37_AllHomopolymers_gt6bp_imperfectgt10bp_slop5.bed.gz
2. “ShortHomopolymer”: grch37.4bp_to_6bp_homopolymers_left_pad_1bp.bed

The gene list used for the patient sample is available at: https://storage.googleapis.com/ur_wgs_public_data/gene_list_example.txt

We used the following publicly available databases for variant filtration and prioritization:
1. NCBI ClinVar 2020-12
2. OMIM 2021-01-06
3. gnomAD release 2.0.2
4. RefSeq Transcripts v91 released 2018-11-09 (Accession No. NM_001001430.1 for patient 1)
We also used the commercially available Qiagen HGMD®Professional Database 2020.4.

The patient sample data cannot be shared under the restrictions placed by the IRB. Source data are provided with this paper.

Field-specific reporting

Please select the one below that is the best fit for your research. If you are not sure, read the appropriate sections before making your selection.

☒ Life sciences ☐ Behavioural & social sciences ☐ Ecological, evolutionary & environmental sciences

For a reference copy of the document with all sections, see nature.com/documents/nr-reporting-summary-flat.pdf

Life sciences study design

All studies must disclose on these points even when the disclosure is negative.

Sample size	No sample size calculation was performed.
Data exclusions	No data were excluded from the analyses.
Replication	We performed 4 replicate sequencing experiments each for barcoded and non-barcoded HG002 data generation and 48 replicate sequencing experiment for the patient samples. All attempts at replication were successful.
Randomization	Randomization was not relevant to our study.
Blinding	Blinding was not relevant to our study

Reporting for specific materials, systems and methods

We require information from authors about some types of materials, experimental systems and methods used in many studies. Here, indicate whether each material, system or method listed is relevant to your study. If you are not sure if a list item applies to your research, read the appropriate section before selecting a response.

Materials & experimental systems

n/a	Involved in the study
☒	☐ Antibodies
☒	☐ Eukaryotic cell lines
☒	☐ Palaeontology and archaeology
☒	☐ Animals and other organisms
☐	☒ Human research participants
☒	☐ Clinical data
☒	☐ Dual use research of concern

Methods

n/a	Involved in the study
☒	☐ ChIP-seq
☒	☐ Flow cytometry
☒	☐ MRI-based neuroimaging

Human research participants

Policy information about studies involving human research participants

Population characteristics	Patient 1: 57 year old male<br>Patient 2: 14 month old female
Recruitment	Enrollment was open to any critical care patient at Stanford hospitals (Stanford Health Care and Lucile Packard Children's Hospital) with a clinical presentation consistent with a genetic disease. Priority was given to patients where a rapidly identified genetic diagnosis would be clinically impactful for the patient or the patient's family. Recruitment of patients was loosely consecutive. While we did not screen all hospital in-patients our study relied on hospital clinicians to contact us in the event that a patient was a potential candidate. We acquired consent from adults directly and for minors, from parents or guardians according to Stanford IRB protocol 58559.
Ethics oversight	Stanford Research Compliance Office - Institutional Review Board [IRB protocol 58559]. The sample consent form has been provided.

Note that full information on the approval of the study protocol must also be provided in the manuscript.
